# Metal Ions Within the Neuro–Immune–Tumor Axis

**DOI:** 10.1155/jimr/1138548

**Published:** 2026-06-29

**Authors:** Xi Cheng, Hong Pan, Yong Dong, Da Li

**Affiliations:** ^1^ The Department of Medical Oncology, Sir Run Run Shaw Hospital Affiliated to Zhejiang University School of Medicine, Hangzhou, 310058, China

**Keywords:** cuproptosis, ferroptosis, metal ions, metallomics, neuro–immune–tumor axis, tumor microenvironment

## Abstract

Metal ions are key regulators of the neuro–immune–tumor axis. Recent studies provide concrete evidence that neuronal Ca^2+^ pulses drive tumor–neuron integration and shape immune signaling. Synaptic Zn^2+^ and ZIP transporters modulate synaptic transmission and tumor growth. Tumor iron accumulation fuels proliferation while creating a clear ferroptosis vulnerability. Copper promotes angiogenesis, lysyl oxidase (LOX)‐mediated extracellular matrix (ECM) remodeling and metastasis, and implicates cuproptosis as a therapeutic target. Mn potentiates cyclic GMP‐AMP synthase (cGAS)–STING signaling and serves as both an immune adjuvant and manganese‐enhanced magnetic resonance imaging (MEMRI) contrast. Besides, Mg and K^+^ regulate kinase/T‐cell receptor (TCR) function and tumor–neuron excitability, respectively. Despite these advances, major gaps persist, notably limited spatial and temporal mapping of labile metal pools, a paucity of cell‐type‐specific causal perturbations, and underdeveloped tumor‐targeted metal‐modulating therapies with proven safety. We therefore propose a focused research agenda: integrate spatial metallomics with single‐cell multiomics, deploy metal‐sensitive longitudinal imaging, apply conditional genetic and chemogenetic perturbations and organotypic models to dissect neuro–immune cross‐talk, and implement preclinical pipelines emphasizing tumor‐selective delivery and comprehensive safety testing.

## 1. Introduction

Metal ions are integral to enzyme catalysis, structural stabilization of macromolecules, mitochondrial respiration, redox balance, and signal transduction [1, 2]. Dysregulated metal homeostasis contributes to neurodegeneration, immune dysfunction, and cancer [3]. Increasingly, metals are recognized as active mediators of intercellular cross‐talk across neurons, immune cells, stromal elements, and cancer cells—an integrated network we refer to as the neuro–immune–tumor axis.

Within the neuronal–immune–tumor axis, metal ions act as biochemical “currencies” that coordinate multicellular responses in the tumor microenvironment (TME) [4]. For instance, Ca^2+^ pulses drive neuronal excitability and are vital for T‐cell activation [5]. Besides, synaptic Zn^2+^ modulates neurotransmission and immune signaling [6]. Meanwhile, Fe and Cu govern mitochondrial metabolism, reactive oxygen species (ROS) production, and regulated cell‐death programs such as ferroptosis and cuproptosis [7, 8]. Tumors actively rewire metal transporters and buffering systems to establish local metal gradients that shape neurotrophic signaling, immune infiltration, and therapeutic responsiveness [9]. Translationally, metal‐related biomarkers (for example, ferritin, ceruloplasmin, and transporter expression), comprehensive metallomic signatures, and metal‐sensitive imaging modalities show promise for diagnosis and treatment monitoring [10].

In addition to their fundamental roles in tumor biology and immune regulation, metal ions have emerged as important therapeutic targets and functional components in cancer nanomedicine [11]. Recent advances in metal‐based nanotherapeutics have enabled the development of catalytic platforms that exploit dysregulated redox homeostasis and metabolic vulnerabilities within the TME. Among these approaches, chemodynamic therapy (CDT) utilizes transition‐metal ions such as Fe and Cu to catalyze Fenton or Fenton‐like reactions, thereby generating toxic ROS selectively in tumor tissues [12]. CDT has been combined with photodynamic therapy, photothermal therapy, chemotherapy, starvation therapy, and immunotherapy to achieve synergistic antitumor effects. Moreover, metal ion‐containing nanoplatforms can modulate ferroptosis, mitochondrial metabolism, and immune activation, further enhancing therapeutic efficacy through TME remodeling and improved antitumor immunity [13]. These findings highlight the growing translational potential of targeting metal‐ion homeostasis and metal‐dependent signaling pathways for precision cancer therapy. Consequently, strategies that modulate metal availability, alter transport, or induce metal‐dependent cell death are under active investigation as potential anticancer and immunomodulatory approaches [14]. Integrating these findings into neuro–immune–tumor research may refine the mechanistic understanding, improve patient selection, and enable rational combination therapies.

In this review, we outline core homeostatic principles and measurement methodologies, then systematically examine Ca^2+^, Zn^2+^, Fe^2+^, Cu^2+^, Mn^2+^, Mg^2+^ and K^+^ across neuronal, immune, and tumor contexts. This synthesis summarizes how metals coordinate neuron–immune–tumor interactions and highlights translational opportunities for diagnostics and targeted therapies. By integrating perspectives from neuroscience, immunology, and oncology, the review bridges disciplinary gaps and provides a unified framework for future research and clinical translation.

## 2. Foundations of Metal Homeostasis in the Microenvironment

Transport, buffering, and storage of metal ions are mediated by specialized importers, exporters, chaperones, and intracellular stores [4]. Calcium homeostasis depends on entry through voltage‐gated calcium channels, TRP channels, and store‐operated Ca^2+^ entry (SOCE), while endoplasmic reticulum stores are controlled by SERCA pumps and IP33R/RYR release channels [15, 16]. For zinc, SLC39/ZIP family members (e.g., ZIP4, ZIP7, and ZIP8) function as importers, while SLC30/ZnT proteins (e.g., ZnT1, ZnT5, and ZnT7) export zinc, and metallothioneins sequester labile Zn^2+^ pools that participate in signaling [17]. Iron uptake is primarily mediated by transferrin receptor 1 (TfR1), which internalizes Fe^3+^–transferrin complexes [18]. DMT1 imports Fe^2+^ across membranes, ferritin stores excess iron, and ferroportin (FPN1) exports Fe^2+^ but is downregulated by hepcidin—an acute phase liver peptide frequently elevated in cancer [19, 20]. Copper uptake occurs via CTR1, after which cytosolic chaperones such as ATOX1 and CCS distribute copper to cuproenzymes [21]. ATP7A and ATP7B traffic copper through the secretory pathway and mediate efflux, processes often altered in chemoresistant tumors [22, 23]. Manganese and magnesium use distinct transporters—Mn^2+^ is handled by DMT1 and ZIP8/ZIP14 [24], whereas Mg^2+^homeostasis involves MAGT1, TRPM6/7, and the CNNM family [24–26]. Reprogramming of these systems in tumors creates spatially heterogeneous metal niches—Fe‐rich, Cu‐rich, or Zn‐depleted regions—that correlate with hypoxia, extracellular matrix (ECM) remodeling, and immune exclusion.

## 3. Metal Ions Across the Neuro–Immune–Tumor Axis

Metal ions are ubiquitous, versatile mediators that shape the communication among neurons, immune cells, and tumor cells within the TME. Acting as metabolic cofactors, second messengers, and structural stabilizers, metals are involved in synaptic transmission, antigen receptor signaling, redox balance, ECM remodeling, and regulated cell‐death programs. Importantly, tumors do not merely adapt to the existing metal landscapes. They actively reconfigure transporter expression and buffering systems to create local metal gradients that promote neurotrophic interactions, suppress or skew immune responses, and alter sensitivity to therapies. In this section, we synthesize current mechanistic insights by metal species, comparing their roles in neuronal physiology, immune function and tumor biology, and highlighting points of intersection where metal‐dependent processes enable neuro–immune cross‐talk (Table 1).

**Table 1:** Metal ions across the neuro–immune–tumor axis.

Metal	Neuronal	Immune	Tumor	Cross‐talk
Ca^2+^	Controls neurotransmission and plasticity [1, 27]; pulses promote glioma via neuroligin‐3 [28].	SOCE (STIM1–ORAI1) in NFAT and cytokine production [29, 30]; CaSR modulates chemotaxis [31].	TRP/VGCC in migration, invasion, and EMT [32, 33]; CaSR linked to aggressiveness [34, 35].	Neuronal Ca^2+^ alters TAMs and T cells [36, 37]; systemic targeting risky; tumor‐selective modulation preferred [38, 39].
Zn^2+^	Synaptic Zn^2+^ modulates NMDA/AMPA/GABA [40, 41]; ZIP7 → ER/Golgi signaling [42, 43].	Required for T cell, DC, and NK function [6, 44]; ZIP/ZnT control signaling pools [17, 45].	Tumors rewire ZIP/ZnT (ZIP4/7) to drive growth [46, 47]; Zn^2+^ stabilizes p53 [48, 49].	Synaptic Zn^2+^ affects immune recruitment [50]; tumor EVs with ZIP4 reprogram microglia [51].
Fe	Essential for ETC and neurotransmitter synthesis [52]; excess → ROS and neuronal injury [53, 54].	Shapes macrophage polarization [55]; affects T cell mitochondrial fitness [56, 57].	Tumors increase TfR1/decrease FPN1 [18, 58]; ferroptosis is a vulnerability [7, 59, 60].	Iron‐rich hypoxic niches drive immune exclusion [61]; ferroptosis impacts antitumor immunity [62].
Cu	Cofactor for enzymes [63]; LOX‐mediated ECM promotes nerve infiltration [64, 65].	Modulates myeloid antigen presentation and T‐cell signaling [66, 67].	Angiogenesis (CTR1/VEGFR2) [68], ECM stiffening [69], metastasis; cuproptosis targets TCA [8, 70].	Cu chelation reduces metastasis [71]; Cu‐PET enables imaging [23]; ECM stiffening fosters immune exclusion [72].
Mn	MEMRI contrast for neuronal activity [73].	Enhances cGAS–STING, boosts type I IFN and antitumor immunity [74, 75].	Functional component in catalytic platforms for synergistic therapy [12, 76].	Mn = immune adjuvant + imaging agent [75]; serum levels nonspecific—use functional assays [10].
Mg	Stabilizes ATP [26]; NMDA receptor “plug” prevents excitotoxicity [27].	MAGT1/TRPM affect TCR and NK receptors [77, 78]; modulates NLRP3 and cytokine maturation [79].	Influences metabolic rate and kinase activity within tumor cells [11].	Repletion and transporter monitoring (e.g., MAGT1) are clinically relevant [25, 78].
K^+^	Critical for neuronal electrophysiology [80]; dysfunction causes hyperexcitability [81].	High extracellular K^+^ in TME suppresses T‐cell effector function [4, 82].	GBM overexpresses channels (e.g., KCND2, EAG2, KCNH2) for invasion and progression [83–85].	Target tumor‐specific K^+^ channels to reduce seizures and tumor growth [83].

### 3.1. Calcium (Ca^2+^)

Ca^2+^ is a central second messenger that coordinates neuronal activity, immune responses, and tumor cell behavior and thus plays a pivotal role in neuro–immune–tumor cross‐talk.

#### 3.1.1. Ca^2+^ in Neuronal Signaling and Tumor Innervation

In neurons, Ca^2+^ orchestrates neurotransmitter release and synaptic plasticity [27]. In glioma models, elevated neuronal activity accelerates tumor growth since optogenetic stimulation increased tumor proliferation through activity‐regulated neuroligin‐3 signaling [28, 86]. Tumor and stromal cells frequently express Ca^2+^‐permeable TRP channels (e.g., TRPC1/6 and TRPV1/4), which promote migration and neurite–tumor coupling [32, 82, 87]. Calcium‐sensing receptor (CaSR) overexpression has been associated with aggressive phenotypes in certain tumors (for example, parathyroid and colorectal metastases), although its clinical utility remains context‐dependent [34, 88, 89]. These findings reveal that Ca^2+^ drives neurotransmission, which modulates the tumor progression.

#### 3.1.2. Ca^2+^ in Tumor Immune Microenvironment

Associations between calcium‐binding genes and tumor mutational burden have been explored [90]. A study reported that the lipid‐lowering agent simvastatin, by modulating Ca^2+^ signaling, enhanced the apoptosis of tumor cells [91]. Another study proposed a novel therapeutic approach that uses photothermal activation of TRPV1 to trigger an intracellular Ca^2+^ cascade [38]. Furthermore, calcium signaling regulates epithelial–mesenchymal transition (EMT) and metabolic reprogramming through pathways such as CaMK and calcineurin [33, 36, 92]. Excessive Ca^2+^ influx via TRP channels and voltage‐gated Ca^2+^ channels (VGCCs) is associated with increased invasion and therapy resistance across multiple cancer types [39, 93]. Collectively, these findings highlight the central role of Ca^2+^ and downstream pathways in tumor progression and therapeutic responses.

Besides, T‐cell receptor (TCR) engagement triggers SOCE via STIM1–ORAI1, which activates the calcineurin–NFAT axis to drive cytokine expression and effector differentiation [29]. Impaired SOCE reduces IL‐2 and IFN‐γ production and diminishes cytotoxicity, underscoring SOCE’s central role in antitumor immunity [94]. In addition, the CaSR plays key roles in immune cells [95]. Originally identified in the parathyroid gland, CaSR is now known to be functionally expressed on T lymphocytes, neutrophils, and monocytes/macrophages [95]. In these immune cells, CaSR regulates multiple functions, including cytokine secretion, chemotaxis, phenotypic switching, and ligand presentation [31, 35]. Dysregulated Ca^2+^ signaling can promote T exhaustion phenotypes within the TME [30]. Targeting Ca^2+^ in solid tumors has been proposed as a therapeutic strategy by enhancing immune‐cell infiltration and accumulation within solid tumor masses.

#### 3.1.3. Ca^2+^ in Neuro–Immune–Tumor Cross‐Talk

Neuronal Ca^2+^ activity controls the release of neurotransmitters and neuropeptides (e.g., glutamate and norepinephrine) that modulate tumor‐associated macrophage (TAM) polarization and T cell infiltration [37, 96]. Conversely, cytokines and ROS produced by immune cells alter neuronal Ca^2+^ homeostasis, reinforcing tumor innervation loops and sustaining protumorigenic signaling [97, 98]. Because systemic Ca^2+^ channel inhibition risks cardioneural adverse effects, tumor‐targeted or local delivery approaches are preferred for therapeutic modulation [99]. Selective targeting of SOCE, TRP channels, or mechanisms of tumor innervation may therefore represent promising strategies to enhance immunotherapy responsiveness by decoupling pathological neuronal–immune–tumor signaling while preserving physiological Ca^2+^ functions.

### 3.2. Zinc (Zn^2+^)

Zinc (Zn^2+^) is a multifunctional ion that modulates neuronal excitability, immune competence, and tumor cell behavior and thereby influences neuro–immune–tumor interactions.

#### 3.2.1. Zn^2+^ in Neurons: Synaptic Zinc and Receptor Modulation

Synaptic Zn^2+^ is coreleased with glutamate at many excitatory synapses and can transiently reach micromolar concentrations, where it modulates NMDA, AMPA, and GABA receptor activity to shape neuronal excitability and synaptic plasticity [40, 42]. Dysregulated zinc transport, particularly ZIP7‐mediated release from the ER/Golgi, rewires downstream signaling cascades, such as ERK and AKT [43], which can contribute to neurogenic inflammation and altered neuronal responses [100].

#### 3.2.2. Zn^2+^ in Immune Cell Function

Zn^2+^, as a signaling ion, regulates both innate and adaptive immunity and influences dendritic cell maturation, macrophage phagocytosis, NK cell cytotoxicity, and T‐cell activation [17, 44]. Zinc is essential for thymic architecture and T‐cell development [101]. Deficiency of zinc impairs adaptive responses, such as reducing CD4+ T‐cell proliferation and lowering IL‐2 production via modulating signaling cascades such as NF‐κB and STAT, shaping antigen‐presenting cell maturation and antitumor immunity [45, 50, 102, 103]. Networks of ZnT and ZIP transporters tightly control intracellular zinc availability and underpin Zn^2+^’s role as a second messenger across immune cell types, as well as zinc’s regulatory roles in TLR, TCR, and cytokine signaling that govern monocyte/macrophage and T‐cell function [104, 105].

#### 3.2.3. Zn^2+^ in Tumor Biology

Elevated ZIP4/ZIP7 expression has been correlated with worse prognosis in some tumor types, suggesting potential utility as tissue biomarkers [46, 47]. Clinical and translational work also links zinc biology to cancer. A study built a seven‐gene ZNF prognostic signature in osteosarcoma associated with immune‐related pathways [106]. The biological effects of Zn^2+^ are highly context‐dependent. Physiological zinc concentrations are required for optimal T‐cell activation and cytokine production. Cancer cells reprogram zinc homeostasis through the altered expression of ZIP and ZnT transporters, affecting proliferation, DNA repair, and apoptosis [104, 107]. Upregulation of transporters such as ZIP4 and ZIP7 promotes oncogenic signaling, while Zn^2+^ binding is critical for stabilizing the DNA‐binding domain of p53; zinc depletion can therefore impair p53‐mediated tumor suppression [48, 49, 108]. Besides, it was found that using multiplex zinc‐finger repressors (ZFRs) to epigenetically silences immune checkpoints and TGFBR2 to augment CAR‐T and TIL activity [109]. Collectively, these findings position Zn^2+^ and zinc‐handling proteins as central regulators of immune function and promising targets for immunomodulatory and anticancer strategies.

#### 3.2.4. Zn^2+^ in Neuro–Immune–Tumor Cross‐Talk

Zinc transporter (ZIP/ZnT) remodeling alters labile Zn^2+^ at synapses and immune synapses, affecting neurotransmission and T‐cell signaling. These coordinated changes establish microenvironmental metal gradients. Synaptically released Zn^2+^ modulates neuronal activity and thereby can influence immune‐cell recruitment and activation [41, 110, 111] in the mouse model. Conversely, zinc availability within the TME shapes tumor‐associated macrophage phenotypes and T‐cell effector function, potentially reinforcing neuro–immune feedback loops [6]. One study shows that ZIP4 uses tumor‐derived EVs to activate the ZIP4–TREM1 axis in glioblastoma, reprogramming the immune microenvironment. Integrating zinc metabolism, immune remodeling, and neurofunctional decline links molecular signaling to local immune changes and global neural dysfunction [51].

Taken together, Zn^2+^ acts as a nodal regulator across the neuro–immune–tumor axis, and context‐specific targeting of zinc transporters or buffering systems may offer therapeutic opportunities. The immunological effects of Zn^2+^ are highly context‐dependent. Physiological zinc concentrations are generally required for optimal T‐cell activation and cytokine production, whereas excessive intracellular zinc accumulation may suppress T‐cell signaling, impair mitochondrial function, or promote apoptosis. Similarly, zinc deficiency compromises thymic development and adaptive immunity, but transient zinc elevation can exert anti‐inflammatory effects by restraining NF‐κB activation. These apparently conflicting findings likely reflect differences in zinc concentration, exposure duration, transporter expression patterns, and immune cell subtype specificity.

### 3.3. Iron (Fe)

Iron (Fe) is a central bioelement whose roles in mitochondrial metabolism, redox biology, immune regulation, and tumor growth make it a key mediator of neuro–immune–tumor interactions.

#### 3.3.1. Fe in Neurons

Iron is essential for electron‐transport chain complexes and neurotransmitter synthesis [52]. Dysregulated Fe elevates ROS via Fenton chemistry, promoting neuronal injury and neuroinflammation that can amplify protumorigenic remodeling and neural‐tumor interactions [112]. Elevated brain iron has also been consistently reported in neurodegenerative disorders such as Alzheimer’s disease and Parkinson’s disease [53], linking iron dysregulation to both neurodegeneration and tumor‐related neural pathology.

#### 3.3.2. Fe in Immune Cells: Macrophage Polarization and T‐Cell Fitness

Iron availability critically regulates immune cell metabolism, differentiation, and effector function through coordinated control of mitochondrial respiration, redox balance, and inflammatory signaling pathways [56]. In the TME, iron‐loaded TAMs preferentially acquire an immunosuppressive and proangiogenic phenotype [55]. Mechanistically, excess intracellular Fe promotes ROS generation through Fenton chemistry, stabilizes HIF‐1 α and NF‐κB signaling, and enhances the expression of ARG1, VEGF, IL‐10, and TGF‐β, thereby driving M2‐like macrophage polarization, angiogenesis, matrix remodeling, and suppression of cytotoxic immunity [55]. Increased ferritin storage and hepcidin‐mediated ferroportin degradation further trap iron within TAMs, reinforcing this suppressive metabolic state. In parallel, tumor cells can exploit macrophage‐derived iron via ferritin uptake and iron‐ export pathways to support proliferation and metastatic adaptation [113]. Clinically, elevated systemic markers of iron handling, including ferritin and hepcidin, correlate with poor prognosis, advanced disease stage, and therapeutic resistance across multiple cancers [114].

Recent work using engineered manganese ferrite nanohybrids showed that iron‐mediated activation of cyclic GMP‐AMP synthase (cGAS)–STING and NF‐κB signaling enhanced inflammatory cytokine production and antigen presentation in macrophages and dendritic cells, promoting polarization toward tumor‐suppressive M1‐like phenotypes characterized by increased CD86 and reduced CD206 expression [76]. Iron‐dependent mitochondrial ROS production further amplified innate immune activation through interferon‐associated signaling pathways. In parallel, tumors can exploit macrophage iron handling to support metastatic progression. Recent evidence demonstrated that metastatic breast cancer cells hijack erythroblastic island macrophages within the bone marrow to acquire iron under hypoxic conditions, thereby promoting tumor survival, bone metastasis, and cancer‐associated anemia [115].

Conversely, insufficient iron availability compromises T‐cell metabolic fitness and antitumor immunity. Iron deficiency reduces the activity of iron–sulfur cluster‐ and heme‐containing mitochondrial electron transport chain complexes, particularly complex II (succinate dehydrogenase) and complex III, leading to impaired oxidative phosphorylation, diminished ATP production, and defective mitochondrial membrane potential [54, 116]. Reduced mitochondrial respiration limits activation‐induced metabolic reprogramming required for rapid T‐cell clonal expansion and cytokine synthesis. Iron restriction also impairs ribonucleotide reductase activity, suppressing DNA synthesis and cell cycle progression, thereby reducing T‐cell proliferation [117]. Functionally, these metabolic defects decrease IFN‐γ, granzyme B, and perforin production, weaken CD8^+^ cytotoxic activity, and promote T‐cell exhaustion [57, 118]. Thus, iron homeostasis exerts bidirectional control over tumor immunity by simultaneously shaping macrophage polarization and sustaining T‐cell energetic competence within the TME.

#### 3.3.3. Fe in Tumors: Ferroptosis, Metabolism, and “Iron Addiction”

Many tumors reprogram iron homeostasis, such as upregulating TfR1 and downregulating ferroportin (FPN1), to increase labile intracellular iron that supports proliferation, EMT, and metastasis [58, 59]. Ferroptosis, an iron‐dependent form of cell death driven by lipid peroxidation, represents a therapeutic vulnerability [60]. For instance, agents such as erastin, RSL3, or GPX4 inhibition reduced tumor volumes in xenograft models depending on the tumor type and dosing [119–121]. Ferroptotic cell death can also modulate antitumor immunity through DAMP release and increased tumor antigenicity [62].

#### 3.3.4. Fe in Axis Cross‐Talk and ROS‐Driven Inflammation

Fe generates ROS that damage lipids, proteins, and DNA. Iron‐driven ROS link neuronal injury to chronic inflammation [122]. Iron‐rich niches frequently overlap with hypoxia and TAM accumulation, creating environments that hinder T‐cell infiltration and promote neural remodeling [61]. Neuronal damage secondary to oxidative lipid injury can both release proinflammatory signals and perturb neuro–immune circuits that normally restrain inflammation [123], creating a feed‐forward loop that promotes tumorigenesis and tumor progression.

### 3.4. Copper (Cu)

#### 3.4.1. Neuronal: Neurotransmitter Enzymes and ECM/Innervation

Copper is essential for several neuronal enzymes, including dopamine β‐hydroxylase and cytochrome c oxidase [63]. Copper‐dependent lysyl oxidase (LOX) crosslinks collagen, stiffens the ECM [64], and thereby facilitates nerve infiltration and tumor metastasis [124]. Through LOX‐mediated ECM remodeling, copper promotes tumor innervation.

#### 3.4.2. Tumor: Cuproenzymes, Angiogenesis, and Cuproptosis

Copper drives angiogenesis via cuproenzymes and proangiogenic signaling [68]. Copper chelation reduced neovascularization and metastasis in preclinical models and showed disease stabilization in subsets clinically [71]. Cuproptosis, a copper‐dependent cell death targeting lipoylated TCA enzymes, reveals vulnerabilities in oxidative tumors [70]. Increased CTR1 and ATP7A/B expression modify copper distribution, supporting cuproenzyme activity (angiogenesis and ECM remodeling) and mediating drug uptake/efflux (e.g., platinum drugs) [125]. Besides, copper modulates ROS generation and affects myeloid functions [126]. Besides, copper availability influences antigen presentation and T‐cell signaling [66, 127], though the effects are context‐dependent.

#### 3.4.3. Axis Cross‐Talk: ECM–Nerve–Immune Coupling

Copper‐dependent cell death pathways also contribute to tumor immune regulation. Copper‐dependent activation of LOX family enzymes promotes oxidative deamination of lysine residues in collagen and elastin, resulting in ECM cross‐linking and matrix stiffening [69]. This biomechanical remodeling enhances integrin‐mediated mechanotransduction, particularly through β1‐integrin/FAK/PI3K–AKT signaling, thereby driving tumor cell survival, migration, and invasive behavior [128]. Increased ECM stiffness also aligns collagen fibers and generates migration tracks that facilitate perineural invasion and metastatic dissemination [129]. Simultaneously, the dense and rigid ECM acts as a physical and biochemical barrier to immune cell trafficking, impairing T‐cell and NK‐cell infiltration and fostering an immune‐excluded TME [72].

Cu‐dependent LOX activity further cooperates with tumor‐secreted sulfated glycosaminoglycan (GAG) pathways [72], which modulate copper availability and matrix organization to reinforce immune evasion and metastatic progression [65]. Disruption of NF‐κB‐mediated copper homeostasis has been shown to sensitize breast cancer cells to cuproptosis, particularly when combined with copper chelation strategies such as tetrathiomolybdate (TTM) [130]. In pancreatic cancer, the SERPINB3–MEK1–MAPK–FOXO3 signaling axis suppresses FDX1 expression, thereby reducing copper‐induced mitochondrial proteotoxic stress and promoting resistance to cuproptosis and antitumor immunotherapy [131]. Another study demonstrated that copper uptake through the transporter SLC31A1 sustains mitochondrial oxidative phosphorylation and NAD^+^/NADH homeostasis in regulatory T (Treg) cells, thereby maintaining peripheral immune tolerance [67]. Independently, another group identified a CD44–hyaluronan‐mediated copper endocytosis pathway in activated macrophages, in which mitochondrial copper accumulation promoted metabolic and epigenetic reprogramming associated with inflammatory signaling activation [66]. In pancreatic cancer, excessive copper accumulation was further shown to promote tumor‐associated neutrophil infiltration and chemoresistance through the activation of the CTR1/TRAF6/STAT3/CCL2 signaling axis [132]. In parallel, dysregulated copper transport machinery—including ceruloplasmin, CTR1 (SLC31A1), and ATP7A/B—has been associated with poor prognosis, enhanced metastatic potential, and resistance to chemotherapy [133].

Collectively, these findings support a coordinated ECM–nerve–immune signaling axis in which copper‐driven stromal remodeling, metabolic rewiring, and immune suppression cooperate to promote neural invasion, immune exclusion, and metastatic dissemination.

### 3.5. Manganese (Mn), Magnesium (Mg), and Potassium (K^+^)

#### 3.5.1. Manganese

Mn^2+^ is a vital regulatory element within the TME that links antitumor immunity with functional imaging. Mechanistically, Mn^2+^ directly enhances cGAS catalytic activity and amplifies STING signaling, robustly boosting type I interferon expression and antitumor immune responses [74]. Consequently, Mn^2+^‐based adjuvants significantly improve preclinical immunotherapy outcomes [75]. Beyond immunomodulation, Mn^2+^ serves as an effective contrast agent in manganese‐enhanced magnetic resonance imaging (MEMRI), allowing longitudinal tracking of neuro‐oncology progression and mapping of active neuronal circuits during tumor infiltration [73]. The indispensability of Mn^2+^ in innate immune surveillance is underscored by the fact that Mn^2+^ ‐deficient mice exhibit accelerated primary tumor growth, enhanced metastasis, and a marked reduction in tumor‐infiltrating CD8^+^ T cells [134]. To exploit these pathways, smart biomaterials have been engineered for targeted delivery. For instance, the manganese‐based nanoadjuvant BMP‐Au couples disulfidptosis‐induced immunogenic cell death (ICD) with Mn^2+^‐mediated cGAS–STING activation to synergistically boost breast cancer immunotherapy [135]. However, strict compartmental dynamics must be maintained as excessive or systemic Mn^2+^ can cause neuroinflammation by inducing mitochondrial defects that amplify inflammasome signaling in microglial cells [136].

Collectively serving as a theranostic bridge across the neuro–immune–tumor axis, Manganese Mn^2+^ orchestrates immune defense via cGAS–STING activation and tracks neuro‐oncology progression through MEMRI, a dual capacity that has driven the design of tumor‐responsive oxide nanomedicines engineered to trigger synergistic ICD while mitigating microglial toxicity.

#### 3.5.2. Magnesium

Mg^2+^ serves as a vital structural and catalytic cofactor that stabilizes ATP and nucleic acids, regulates kinase and phosphatase activities, and critically modulates TCR signaling dynamics [77]. At the molecular level, a reduction in extracellular Mg^2+^ decreases intracellular concentrations, directly impairing downstream Ca^2+^ flux, activation marker upregulation, and T‐cell proliferation following TCR stimulation. Mechanistically, Mg^2+^ deficiency selectively subverts TCR signal transduction via the IL‐2‐inducible T‐cell kinase (ITK) due to the absolute requirement of a regulatory Mg^2+^ ion within its catalytic pocket [77]. Furthermore, Mg^2+^ availability governs the function of the costimulatory cell‐surface integrin LFA‐1 on CD8^+^ T cells; Mg^2+^ binding induces the active conformation of LFA‐1, which subsequently augments antigen‐specific cytotoxicity [137]. Beyond direct lymphocytic activation, Mg^2+^ modulates NLRP3 inflammasome priming and subsequent cytokine maturation [79], processes that can be deregulated by persistent inflammation during early‐stage disease [138]. Conversely, genetic loss‐of‐function models demonstrate that MAGT1, the Mg^2+^ transporter, deficiency selectively disrupts these pathways, causing impaired NKG2D expression and recurrent infections [78].

Reflecting these profound immunoregulatory functions, systemic magnesium homeostasis has clinical relevance during cancer immunotherapy. In patients undergoing immune checkpoint blockade therapy, those with elevated baseline serum magnesium levels exhibit significantly superior clinical outcomes and favorable prognoses compared to those of their hypomagnesemic counterparts [139]. Driven by these clinical insights, advanced biomaterial platforms have been designed to manipulate the tumor immune microenvironment through targeted ion delivery. For instance, a dual‐loading hydrogel platform achieves the precise, spatiotemporally regulated discharge of Mg^2+^, successfully reversing local immunosuppression and augmenting the efficacy of systemic ICB therapy [140]. Beyond its localized immunomodulatory applications, perioperative Mg^2+^ homeostasis is also implicated in systemic physiological stabilization. Rotational thromboelastometry (ROTEM) analysis indicates that intraoperative administration of intravenous magnesium sulfate significantly attenuates blood hypercoagulability in patients undergoing laparoscopic surgery for colorectal cancer [141], suggesting a broader protective utility across the perioperative oncology continuum.

#### 3.5.3. Potassium

Research shows that potassium channels are critical for neuronal electrophysiological stability [80]. Their dysfunction causes neuronal hyperexcitability [81]. In the GBM microenvironment, tumor‐secreted cytokines and bioactive factors can disrupt potassium channel regulation in nearby neurons, upsetting the excitability balance. In GBM and tumor‐associated epilepsy, oligodendrocyte‐progenitor‐like (OPC‐like) tumor cells accumulate at the tumor–neuron interface and overexpress KCND2. The Kv4.2 channel encoded by KCND2 promotes extracellular K^+^ accumulation and increases neuronal excitability [83]. Additionally, EAG2 localizes to tumor–neuron contact sites in a Kvβ2‐dependent manner; genetic disruption of the EAG2–Kvβ2 complex reduces GBM calcium transients, limits tumor growth and invasion, and prolongs survival in tumor‐bearing mice [84], supporting the complex as another therapeutic candidate. More importantly, Ca^2+^ can activate K^+^ channels in the GBM. Modulation of the activity of the Ca^2+^‐activated K channels reduced the proliferation and migration of GBM cells and suppressed glioma progression in both in vivo and in vitro cell models for GBM [142]. Another study revealed that KCNH2, a gene associated with voltage‐gated potassium channels, is differentially expressed in more than 30 types of cancer and exhibits high diagnostic value for 10 tumor types, which is associated with poor prognosis in GBM and hepatocellular carcinoma [85]. Currently, treatments involving K ions are being developed. Encapsulated within ApoE‐peptide‐modified liposomes, it significantly inhibits the growth of xenografted GBM, reduces cardiotoxicity, and enables in vivo imaging [143]. In summary, these findings suggest that targeting K^+^ channels is a potential approach for treating cancers such as GBM.

**Figure 1:**
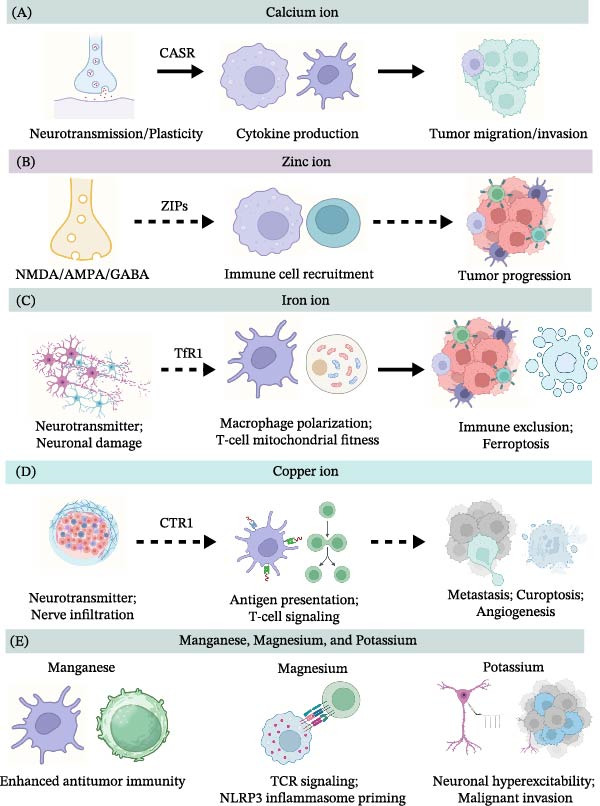
Schematic summary of metal ion‐mediated signaling across the neuro–immune–tumor axis. Divalent metal ions within the tumor microenvironment (TME) function as critical messengers coordinating the cross‐talk between neuronal circuits, immune populations, and malignant cells. (A) Calcium (Ca^2+^): neuronal Ca^2+^ activity and synaptic pulses drive tumor integration via neuroligin‐3 signaling. Simultaneously, SOCE‐mediated Ca^2+^ flux regulates NFAT activation in T cells, while Ca^2 **+**^ gradients in the TME facilitate tumor cell migration and invasion. (B) Zinc (Zn^2 **+**^ ): synaptic Zn^2 **+**^ release modulates glutamatergic and GABAergic neurotransmission. In the TME, tumor‐derived extracellular vesicles (EVs) utilize Zn^2 **+**^ transporters (e.g., ZIP4) to reprogram microglial plasticity and drive progression. (C) Iron (Fe): iron hoarding by tumor cells (via high TfR1/low FPN1) limits availability for T cell mitochondrial fitness [56]. Dysregulated iron metabolism promotes immunosuppressive macrophage polarization (M2‐like) and creates a niche characterized by ROS‐driven neuronal injury and ferroptosis vulnerability. (D) Copper (Cu^2 **+**^ ): copper facilitates nerve infiltration via LOX‐mediated ECM remodeling. It further drives angiogenesis and metastasis through CTR1/VEGFR2 signaling, while high mitochondrial copper levels sensitize cells to cuproptosis by targeting lipoylated TCA cycle proteins. (E) Manganese, magnesium, and potassium (Mn^2 **+**^, Mg^2 **+**^, K ^**+**^ ): Mn^2 **+**^ acts as a potent adjuvant by activating the cGAS–STING pathway to boost antitumor immunity [75]. Mg^2 **+**^ (via MAGT1) is essential for TCR signaling and NLRP3 inflammasome priming [78], while K ^**+**^ channel overexpression in tumors (e.g., EAG2) drives both neuronal hyperexcitability and malignant invasion [83].

Taken together, these metal ions act as central mediators of the neuro–immune–tumor axis by coordinating neuronal activity, immune responses, and cancer cell behavior within the TME (Figure 1). Neuronal Ca^2+^ and synaptic Zn^2+^ shape neurotransmission and tumor innervation; iron and copper reprogram metabolism, redox state, and ECM remodeling to favor tumor growth, immune exclusion, and metastatic spread; manganese potentiates cGAS–STING signaling and serves as an immune adjuvant/imaging agent, while magnesium and potassium regulate kinase/TCR function and neuronal excitability, respectively. Tumors actively rewire metal transporters and buffering systems to create spatially heterogeneous metal niches that influence macrophage polarization, T‐cell fitness, antigen presentation, and regulated cell‐death programs.

### 3.6. Future Directions and Conclusions

The current findings reveal that the metal ions act as critical mediators at the intersection of neuronal activity, immune function, and tumor biology. While individual metal‐specific mechanisms have been delineated, major gaps remain in understanding spatial and temporal metal dynamics across cell types within the TME and their causal roles in therapy response and neurological comorbidity. The following priorities and experimental strategies aim to translate mechanistic insights into clinically actionable biomarkers and safer, targeted interventions.

Advances in single‐cell and spatial metallomics, integrated with transcriptomics and proteomics, should be scaled to produce high‐resolution, cell‐type‐specific metal atlases in vivo [144–146]. Combining techniques such as LA‐ICP‐MS/XRF with single‐cell RNA‐seq and proteomic profiling will reveal which cell populations harbor labile versus stored metal pools, map metal‐dependent signaling circuits, and resolve spatial relationships among metal gradients, nerve terminals, and immune infiltrates [147, 148]. These multimodal maps will be critical for identifying actionable hotspots and for informing where and when to intervene in the TME.

To establish causality, targeted genetic dissection across the neuronal, immune, and tumor compartments is required. Conditional, cell‐type‐specific perturbations (for example, knockout/knockdown of CTR1, FPN1, ZIP7, or the STIM1–ORAI1 axis) paired with functional readouts (ferroptosis/cuproptosis sensitivity, STING activation, electrophysiology, and behavioral assays) will link transporter activity and metal handling to downstream phenotypes such as tumor growth, immune evasion, and neuro–immune cross‐talk [149]. Such causal models also provide rigorous platforms for testing therapeutic hypotheses and safety signals before clinical translation.

On the translational side, biomarker‐driven clinical trials and improved metal‐sensitive diagnostics are priorities for tumor progression. Despite increasing interest in metallomic signatures and transporter expression profiles, clinically validated biomarkers capable of reliably predicting immunotherapy responsiveness remain limited. Candidate biomarkers, including serum Cu/Fe ratios, ferritin, ceruloplasmin, hepcidin, CTR1 expression, and ZIP‐family transporter profiles, have shown associations with prognosis or immune phenotypes in exploratory studies, but reproducibility across tumor types and patient cohorts remains insufficient for routine clinical implementation. Future biomarker development should integrate metallomic profiling with immunophenotypic parameters such as T‐cell exhaustion markers, macrophage polarization states, and spatial immune cell distribution to improve predictive performance and patient stratification. Trials should stratify patients by metallomic signatures and transporter expression rather than by tissue of origin alone and should embed metal imaging (e.g., Cu‐PET and MEMRI) and spatial metallomics on biopsy or resection specimens to confirm target engagement [10]. Parallel efforts must validate composite liquid‐biopsy panels (plasma/exosome metal profiles plus ferritin, hepcidin, ceruloplasmin, and transporter ectodomains) in multicenter cohorts with strict preanalytical SOPs to control trace‐metal contamination, fasting/hemolysis effects, and assay limits of detection.

Several metal‐modulating strategies have entered clinical or translational evaluation. TTM, a copper‐chelating agent initially developed for Wilson disease, demonstrated antiangiogenic activity and disease stabilization in subsets of metastatic breast cancer patients in early‐phase clinical studies [150]. Furthermore, animal studies have shown that TTM aids the excretion of copper via the biliary tract, while human studies have found that TTM reduces the absorption of copper in the gut [151]. Likewise, deferasirox, which has already received clinical approval, is currently being investigated for its effects on brain iron metabolism in patients with aneurysmal subarachnoid hemorrhage. Furthermore, deferasirox and deferoxamine are currently being studied for use in combination therapy to exploit tumors’ dependence on iron and their sensitivity to ferroptosis [152, 153]. A major translational challenge is the incomplete safety characterization of systemic metal‐modulating therapies. Excessive manipulation of iron or copper metabolism may induce hepatotoxicity, nephrotoxicity, neurotoxicity, hematologic suppression, or immune dysregulation due to the disruption of physiological metal homeostasis. Therefore, future translational efforts should prioritize TME‐responsive delivery systems, including pH‐sensitive copper chelators, ROS‐responsive nanoparticles, and hypoxia‐activated iron carriers, to improve tumor selectivity and minimize systemic toxicity. In parallel, integrated biomarker platforms combining metallomic signatures with immunophenotypic profiling, such as serum metal‐ion ratios, transporter expression, T‐cell subset distribution, and macrophage polarization states, may enable patient stratification, treatment monitoring, and prediction of immunotherapy responsiveness.

In conclusion, metal ions are central nodes linking neuronal signaling, immune regulation, and tumor biology. Advances in ferroptosis, cuproptosis, transporter biology, and spatial metallomics are opening up diagnostic and therapeutic avenues.

## Author Contributions

Conception, design, and manuscript preparation: Xi Cheng. Figure preparation: Hong Pan and Yong Dong. Approval of the final manuscript and fund acquisition: Da Li.

## Funding

This work was supported by the grant from the Zhejiang Provincial Natural Science Foundation (Grant LY24H160004) and the Clinical Medical Research Special Fund of Zhejiang Medical Association (Grant 2022ZYC‐D08).

## Disclosure

All authors have given their consent to publication. Da Li approved the final manuscript.

## Ethics Statement

The authors have nothing to report.

## Conflicts of Interest

The authors declare no conflicts of interest.

## Data Availability

Data sharing is not applicable to this article, as no datasets were generated or analyzed during the current study.
